# 16S rRNA deep sequencing identifies *Actinotignum schaalii* as the major component of a polymicrobial intra-abdominal infection and implicates a urinary source

**DOI:** 10.1099/jmmcr.0.005091

**Published:** 2017-05-03

**Authors:** Andrew Bryan, Lindsey M. Kirkpatrick, John J. Manaloor, Stephen J. Salipante

**Affiliations:** ^1^​ Department of Laboratory Medicine, University of Washington, Box 357110, 1959 NE Pacific Street, NW120, Seattle, WA, USA; ^2^​ Ryan White Center for Pediatric Infectious Diseases and Global Health, Department of Pediatrics, Indiana University School of Medicine, Indianapolis, IN, USA

**Keywords:** polymicrobial, *Actinotignum shaalii*, 16S rRNA, deep sequencing, abdominal, urinary tract infection

## Abstract

**Introduction.** It can be difficult to catalogue the individual organisms comprising polymicrobial patient infections, both because conventional clinical microbiological culture does not facilitate the isolation and enumeration of all members of a complex microbial community, and because fastidious organisms may be mixed with organisms that grow rapidly *in vitro*. Empiric antimicrobial treatment is frequently employed based on the anatomical site and the suspected source of the infection, especially when an appropriately collected surgical specimen is not obtained.

**Case presentation.** We present a case of an intra-abdominal infection in a patient with complex anatomy and recurrent urinary tract infections. Imaging did not reveal a clear source of infection, no growth was obtained from urine cultures and initial abdominal fluid cultures were also negative. In contrast, 16S rRNA deep sequencing of abdominal fluid samples revealed mixed bacterial populations with abundant anaerobes, including *Actinotignum schaalii* (*Actinobaculum schaalii*). Ultimately, only *Enterobacter cloacae* complex and meticillin-resistant *Staphylococcus aureus*, both of which were identified by sequencing, were recovered by culture.

**Conclusion.** The clinical application of 16S rRNA deep sequencing can more comprehensively and accurately define the organisms present in an individual patient's polymicrobial infection than conventional microbiological culture, detecting species that are not recovered under standard culture conditions or that are otherwise unexpected. These results can facilitate effective antimicrobial stewardship and help elucidate the possible origins of infections.

## Abbreviations

CT, computed tomography; HD, hospital day; UTI, urinary tract infection; WBC, white blood cell.

## Introduction

Complex intra-abdominal infections pose both diagnostic and therapeutic dilemmas given their frequent polymicrobial nature [1, 2]. Empiric, broad-spectrum therapy is recommended, with adjustments if unusual organisms or resistant isolates are identified [1, 3]. However, culturing fastidious and/or anaerobic organisms from such infections can be challenging, and may be confounded by co-infection with organisms that grow rapidly *in vitro* [4–6]. Here, we report an intra-abdominal infection marked by a complex, polymicrobial community containing *Actinotignum schaalii* (*Actinobaculum schaalii*) as the major species. This fastidious organism was detected by 16S rRNA deep sequencing, whereas only *Enterobacter cloacae* complex and *Staphylococcus* *aureus*, which were lesser components of the infectious process, were identified by culture-based methods.

## Case report

**Fig. 1.**
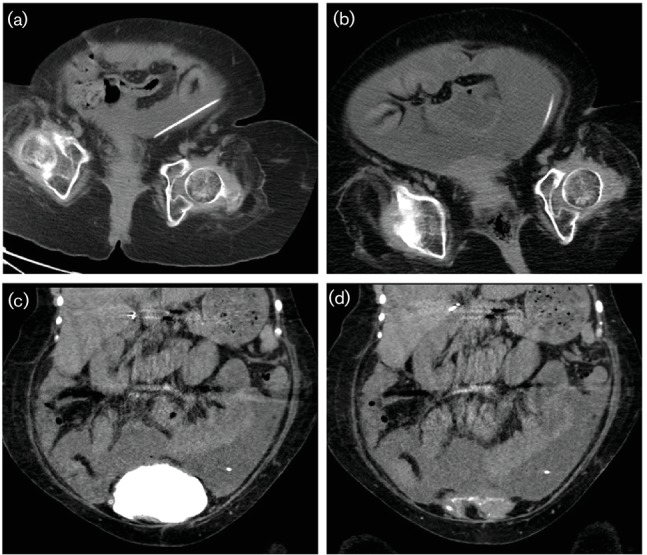
(a, b) Axial cross-section from CT of the abdomen and pelvis demonstrating: small volume pelvic fluid collection 1 year prior to the illness (a) and large pelvic fluid collection at the time of admission (b). (c, d) Coronal cross-section from contrasted CT cystogram demonstrating no detectable extravesicular dye in the abdomen and pelvis on admission: pre-void (c) and post-void (d).

A 21-year-old woman with a history of myelomeningocele, ventriculoperitoneal shunt, and neurogenic bowel and bladder presented to an outside hospital with acute worsening of a 1 year history of intermittent abdominal pain and distension. Her history was significant for remote Malone anterograde continence enema and Monti procedures to attain faecal and urinary continence (requiring frequent catheterization), recurrent urinary tract infections (UTIs), and bladder perforation and repair. She was diagnosed with a UTI and discharged on ciprofloxacin. Urinalysis at the time of the initial presentation to the outside hospital revealed: specific gravity 1.025; pH 6; hazy; trace ketones and proteins; small leukocyte esterase; 20–30 white blood cells per high powered field; 10–20 red blood cells per high powered field; positive for nitrites; moderate bacteria; mucous; and no glucose/urobilinogen present. Routine urine cultures were ultimately negative. Her symptoms worsened over the following 2 days and she presented to a paediatric hospital, where she was found to be diaphoretic with middle left quadrant and epigastric abdominal pain and a distended abdomen. Laboratory test results were notable for leucocytosis (18 500 cells mm^−3^) without left shift and a urinalysis that was not concerning for infection (pH 6, negative for nitrites and leukocyte esterase, and no microscopic evidence of WBCs or red blood cells). Urine cultures were collected: blood agar and MacConkey plates (Remel) were incubated for at least 18 h at 35 °C under ambient air and were finalized as no growth. Antimicrobials were initially stopped at the paediatric hospital given the initial urine studies did not indicate a UTI, lack of fever, stable vital signs and baseline abdominal distension. Ciprofloxacin, which she had taken inconsistently as an outpatient, was discontinued. A computed tomography (CT) cystogram and CT scan of the pelvis (Fig. 1) and an ultrasound (not shown) demonstrated complex abdominal fluid collection, but no evidence of bladder perforation.

Three days after admission to the paediatric hospital [hospital day (HD) 3], a percutaneous drain was put in place. Fluid studies revealed 1010 nucleated cells ml^−1^ (97 % neutrophils), <0.5 g albumin dl^−1^, <10 units amylase l^−1^, 38 mg glucose dl^−1^, 460 units lactate dehydrogenase l^−1^ and 0.2 mg fluid creatinine dl^−1^. Gram stain showed 1+ WBC, but no organisms. Abdominal fluid cultures were plated on blood, chocolate, MacConkey, CNA (colistin and nalidixic acid), PEA (phenylethyl alcohol) and anaerobic blood agar media (Remel). The patient was administered piperacillin/tazobactam and then transitioned to cefepime on HD14 after recurrent fever and isolation of *E. cloacae* complex, as described below. Upon the switch to cefepime, metronidazole was added for improved anaerobic coverage. Aerobic (5–10 % CO_2_) and anaerobic abdominal fluid cultures remained negative after 5 days of incubation at 35 °C.

The patient’s ventriculoperitoneal shunt was externalized on HD5. She was started on vancomycin on HD9 after developing pustules around her abdominal incision site. Repeat CT cystogram showed a rim enhancing pelvic fluid collection and a Malone anterograde continence enema fluoroscopic study showed a patent colon without stricture or urethral fistula. Her abdominal drain was exchanged on HD10, at which time fluid was again submitted for culture on solid media. Abdominal fluid was also inoculated at the bedside into aerobic and anaerobic blood culture bottles (BD BACTEC Plus) and sent for broad-range bacterial 16S rRNA PCR (Clinical Microbiology Laboratory, University of Washington, Seattle, WA, USA).

Conventional 16S rRNA Sanger sequencing [7] resulted in an uninterpretable chromatogram, suggestive of multiple bacterial templates. Therefore, 16S rRNA deep sequencing (Illumina MiSeq) was performed with data processing and sequence classification as previously described [4, 6, 8]. A total of 4 94 324 raw sequence reads were obtained. Taxonomic classification by deep sequencing identified multiple organisms, with the highest number of reads corresponding to *A. schaalii* (Table 1). *E. cloacae* complex grew from both the aerobic and anaerobic blood culture bottles, while meticillin-resistant *S. aureus* grew from the aerobic blood culture bottle. Solid media did not recover any organisms after 5 days of incubation.

**Table 1.** Organisms identified by 16S rRNA deep sequencing

Reads	Per cent of total reads*	Per cent identity	Assignment
91 897	34.09	99.03–100.0	*Actinotignum schaalii*
2 02 293	30.53	99.01–100.0	Bacteria of family *Enterobacteriaceae*†
38 800	10.8	97.22–100.0	*Peptoniphilus* species related to *Peptoniphilus indolicus*
35 435	7.89	99.01–100.0	*Staphylococcus aureus*
23 612	5.84	99.02–100.0	*Campylobacter ureolyticus*
16 970	4.72	99.08–100.0	*Helcococcus kunzii*
12 852	3.58	99.04–100.0	*Finegoldia magna*
4 738	1.27	97.03–99.34	*Anaerococcus* species

*Percentage of total reads corrected for abundance of rRNA sequences; only organisms with abundances >1 % are shown.

†The 16S rRNA V1-V2 region does not permit reliable species-level classifications within the family *Enterobacteriaceae*.

The patient underwent tPA (tissue plasminogen activator)-mediated lysis of her fluid collection and was discharged after 21 days on vancomycin, cefepime and metronidazole, eventually completing 45 days of therapy with resolution of signs and symptoms.

## Discussion

Approximately 3 in 10 000 live births are affected by spina bifida, with the majority having neurogenic bladder [9]. Over 40 % of those patients will have >5 UTIs by age 15 years [9–12]. The complex anatomy, procedural history and antimicrobial exposure in our patient further increased her risk for UTI with a broad range of pathogens that may differ from uncomplicated community-acquired infections [9–12]. Imaging and fluid chemistry studies, including creatinine, did not implicate a specific source of infection. The differential was consequently broad and included the cerebrospinal fluid /shunt, genitourinary system and gastrointestinal tract.

Although only *E. cloacae* complex and meticillin-resistant *S. aureus* were isolated by culturing patient specimens; 16S rRNA deep sequencing enumerated a polymicrobial population (Table 1), which was predominantly composed of *A. schaalii*. In total, six of the eight organisms identified by sequencing were not recovered by conventional techniques. These results are consistent with controlled experiments, which have indicated that standard culture conditions either fail to recover or significantly distort the relative abundance of organisms present in polymicrobial samples [4]. *A*. *schaalii*, in particular, is challenging to grow and to phenotypically characterize [13]. Although the standard urine culture conditions used in this case would not have recovered *A*. *schaalii*, the organism can be isolated on blood agar plates incubated at 5 % CO_2_ for 48 h [13, 14]. This suggests that culture of the body fluid could have supported growth of the organism, but that the organism was either non-viable at the time of sampling due to prolonged antimicrobial exposure or was outcompeted *in vitro* by the more rapidly growing *S. aureus* or *E. cloacae*.

The detection of *A*. *schaalii* was suggestive of a urinary aetiology for the patient’s infection, possibly a bladder microperforation [13, 15, 16]. In support of this hypothesis, most of the additional organisms recovered by 16S rRNA sequencing have also been reported in varying abundances in urine [15, 17–21]. The normal fluid creatinine suggests a time frame sufficient for autodialysis of extravasated urine, and supports the subacute to chronic onset of infection rather than an acute urological pathology [22, 23]. However, given the patient’s anatomy, it is also possible that skin and gastrointestinal microbiota were contributory.

*A. schaalii* has been an infrequently described, but likely under-recognized cause of UTIs and abdominal abscesses[13]. Despite studies showing a prevalence in urine of greater than 15 % by PCR [24], it is rarely isolated in urine cultures given its slow growth (~48 h) and a requirement for low-oxygen conditions. The organism’s identification has also been historically complicated by challenges with biochemical identification methods, although this has been remedied in laboratories that have adopted matrix-assisted laser desorption ionization-time of flight mass spectrometry [13, 14, 25, 26]. The human ecological niche appears to be primarily the genitourinary system [27]. Although typically susceptible to β-lactams and vancomycin, *A. schaalii* is frequently resistant to macrolides, clindamycin, trimethoprim/sulfamethoxazole, metronidazole and fluoroquinolones [13, 28]: many of the agents typically used to treat UTIs and for potential de-escalation of therapy for complex intra-abdominal infections. In this case, the anaerobes identified by sequencing are typically susceptible to β-lactams, while resistance rates to clindamycin may be up to 40 % for *Peptoniphilus* and *Finegoldia magna* [19, 29]. Of note, *Bacteroides* spp., typically among the most drug-resistant anaerobic organisms, were not identified (Table 1) [29]. De-escalation of antimicrobials was challenging in this case given the simultaneous identification of meticillin-resistant *S. aureus* and *Enterobacter*; however, source control and β-lactam therapy should have been sufficient for the anaerobes identified [29].

This case demonstrates the utility of 16S rRNA deep sequencing in patient management. Organisms with specific ecological niches and microbiological culture requirements were identified far more comprehensively by sequencing, but were not recovered by standard clinical culture, providing insight into the aetiology of the patient’s infection. To our knowledge, the application of 16S rRNA deep sequencing or metagenomics of clinically infected individual patient samples has not been previously described as a tool to help determine the route of a patient’s infection. Even though many fastidious organisms, such as *A*. *schaalii*, can be recovered using sufficiently broad culture conditions [30], it is not possible to anticipate the relevant organisms in all specimens and there are practical limits to the number of plates that can be applied per specimen. High-throughput sequencing is inherently scalable, and able to recover species unbiasedly and without prior expectation [4]. Moreover, accurate characterization of polymicrobial infections can facilitate appropriate escalation or de-escalation of therapy, while minimizing exposure to broad-spectrum antimicrobials and supporting antibiotic stewardship. Further integration of high throughput 16S rRNA sequencing with clinical practice has the potential to improve patient care [4].
